# Non-hyalinizing trabecular thyroid adenoma: a novel thyroid tumor with diagnostic pitfalls of hyalinizing trabecular adenoma and medullary thyroid carcinoma

**DOI:** 10.1186/s13000-023-01361-5

**Published:** 2023-06-20

**Authors:** Mitsuyoshi Hirokawa, Michiko Matsuse, Norisato Mitsutake, Ayana Suzuki, Miyoko Higuchi, Toshitetsu Hayashi, Hiroshi Kamma, Akira Miyauchi, Takashi Akamizu

**Affiliations:** 1 Department of Diagnostic Pathology and Cytology, Kuma Hospital, 8-2-35 Shimoyamate-Dori, Chuo-Ku, Kobe, Hyogo 650-0011 Japan; 2 Department of Radiation Medical Sciences, Atomic Bomb Disease Institute, Nagasaki University, Nagasaki, Japan; 3 Nasu Insititute of Medical Sciences, Nasushiobara, Japan; 4 Department of Surgery, Kuma Hospital, Kobe, Japan; 5 Department of Internal Medicine, Kuma Hospital, Kobe, Japan

**Keywords:** Thyroid, Hyalinizing trabecular tumor, Medullary thyroid carcinoma, *PAX8/GLIS3* fusion gene

## Abstract

**Background:**

Only one thyroid follicular cell-derived tumor with a purely trabecular growth pattern has previously been described. This report aims to describe the histological, immunohistochemical, and molecular findings of our second case, propose a novel thyroid tumor, and discuss its diagnostic pitfalls.

**Case presentation:**

A 68-year-old female presented with an encapsulated thyroid tumor composed of thin and long trabeculae. No papillary, follicular, solid, or insular patterns are observed. The tumor cells were elongated or fusiform and arranged perpendicular to the trabecular axis. No nuclear findings of papillary thyroid carcinoma and increased basement membrane material were found. Immunohistochemically, the tumor cells were positive for paired-box gene 8, thyroid transcription factor-1, and negative for thyroglobulin, calcitonin, and chromogranin A. Inter- and intra-trabecular accumulation of type IV collagen-positive materials was not demonstrated. None of *PAX8/GLIS1 and PAX8/GLIS3 and mutations in BRAF, HRAS, KRAS, NRAS, TERT promoter, CTNNB1, PTEN, *and *RET* were detected.

**Conclusions:**

We report our case as a novel disease entity called non-hyalinizing trabecular thyroid adenoma, which has the diagnostic pitfalls of hyalinizing trabecular tumor and medullary thyroid carcinoma.

## Background

Among thyroid tumors, follicular and papillary growth patterns are the most frequently observed. A trabecular growth pattern is characteristic of hyalinizing trabecular tumor (HTT) and is also present in papillary thyroid carcinoma (PTC), follicular thyroid adenoma (FTA), follicular thyroid carcinoma (FTC), poorly differentiated thyroid carcinoma (PDTC), and medullary thyroid carcinoma (MTC) [1]. Thyroid tumors with trabecular growth patterns are usually mixed with other growth patterns. Recently, we encountered a case of a thyroid follicular cell-derived tumor that was composed of a purely trabecular growth pattern and histologically required differentiation from HTT or MTC. To the best of our knowledge, only one case with the unique histological findings has previously been described as a purely trabecular follicular adenoma of the thyroid [2]. Molecular testing was not performed in the case. This report aims to describe the histological, immunohistochemical, and molecular findings of our second case, propose a novel thyroid tumor, and discuss its diagnostic pitfalls.

## Case presentation

**Fig. 1:**
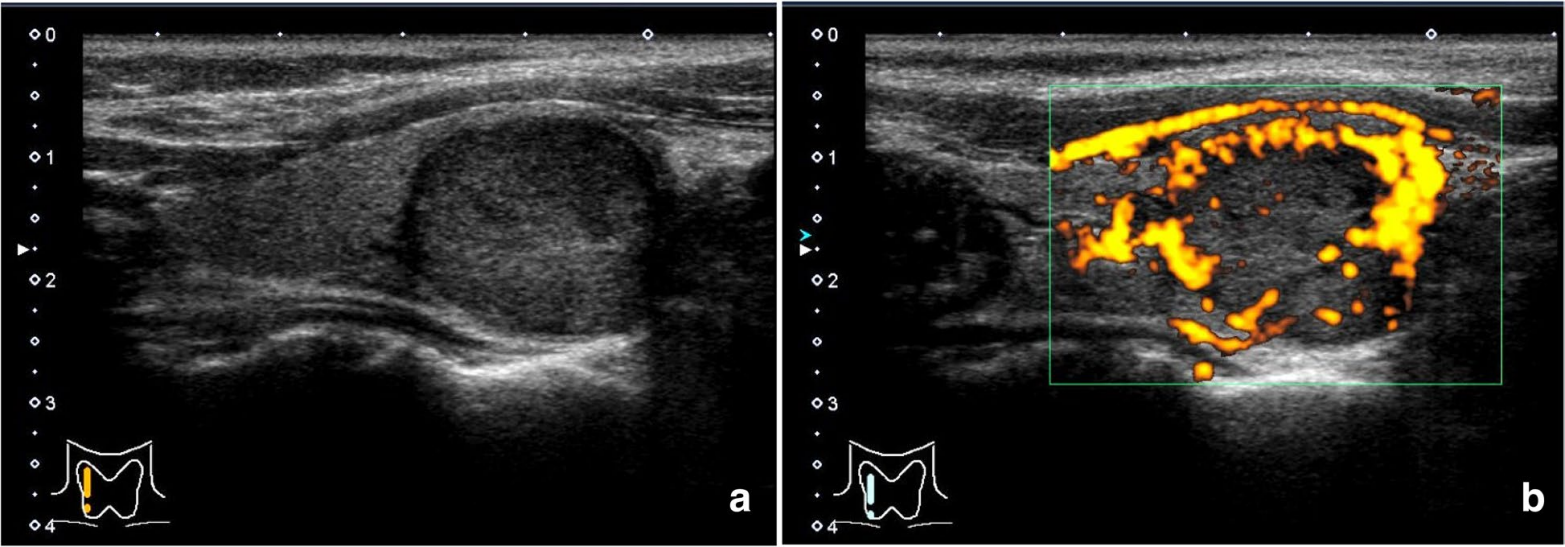
A well-defined, hypoechoic, and oval mass is present in the right lobe (**a**). Doppler imaging reveals increased peritumoral blood flow (**b**) (B-mode)

A 68-year-old female with a thyroid mass was referred to our hospital. Thyroid function tests, including thyroid-stimulating hormone, free T4, free T3, and thyroglobulin, were within normal limits. Ultrasound examination revealed a well-defined, hypoechoic, and oval mass measuring 20 × 17 × 18 mm (Fig. 1). It had a homogeneous echo texture and was not associated with any calcification. No peripheral halo signs were present. Doppler imaging revealed an increase in peritumoral blood flow. No so-called “tumor inferno” was found. No lymph node swelling was observed in the neck. This was interpreted as a follicular tumor.

**Fig. 2:**
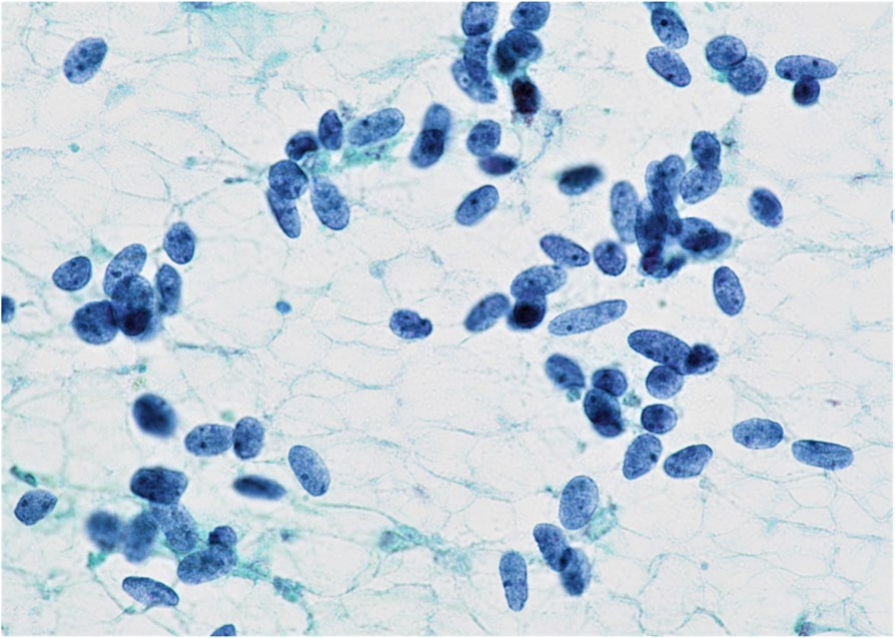
Tumor cells appear as naked nuclei. The nuclei are short-spindled in shape and show a granular chromatin pattern (Papanicolaou stain)

Ultrasound-guided fine-needle aspiration cytology revealed moderate cellularity, composed of discohesive and naked cells. The nuclei were short-spindled in shape and bland (Fig. 2). The nuclei showed a granular chromatin pattern and small nucleoli. No nuclear features characteristic of PTC, such as intranuclear cytoplasmic inclusions, nuclear grooves, or powdery chromatin, were observed. The cytology report was “malignant; MTC.” However, the serum calcitonin and CEA levels (< 0.05 pg/mL, 4.3 ng/mL) showed no increase; moreover, the calcitonin stimulation tests were unresponsive (3 min 6.46 pg/mL, 5 min 4.99 pg/mL). The patient had no family history of MTC, and germline *RET* mutations were not detected. To make the diagnosis and treatment, a subsequent right lobectomy was performed.

**Fig. 3:**
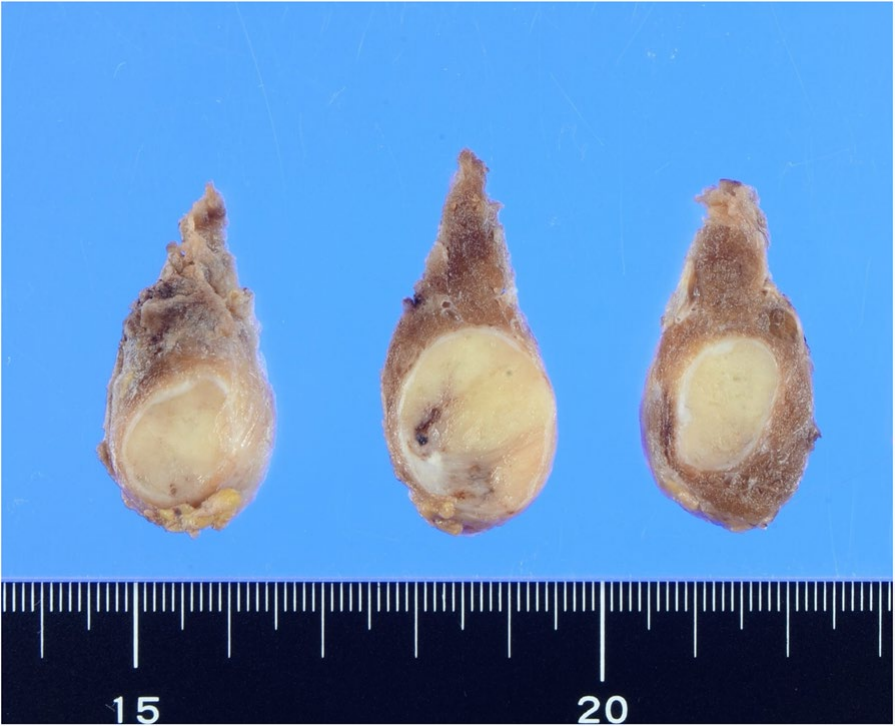
The mass is encapsulated by a thick capsule. The cut surface is solid, whitish-yellow in color

**Fig. 4:**
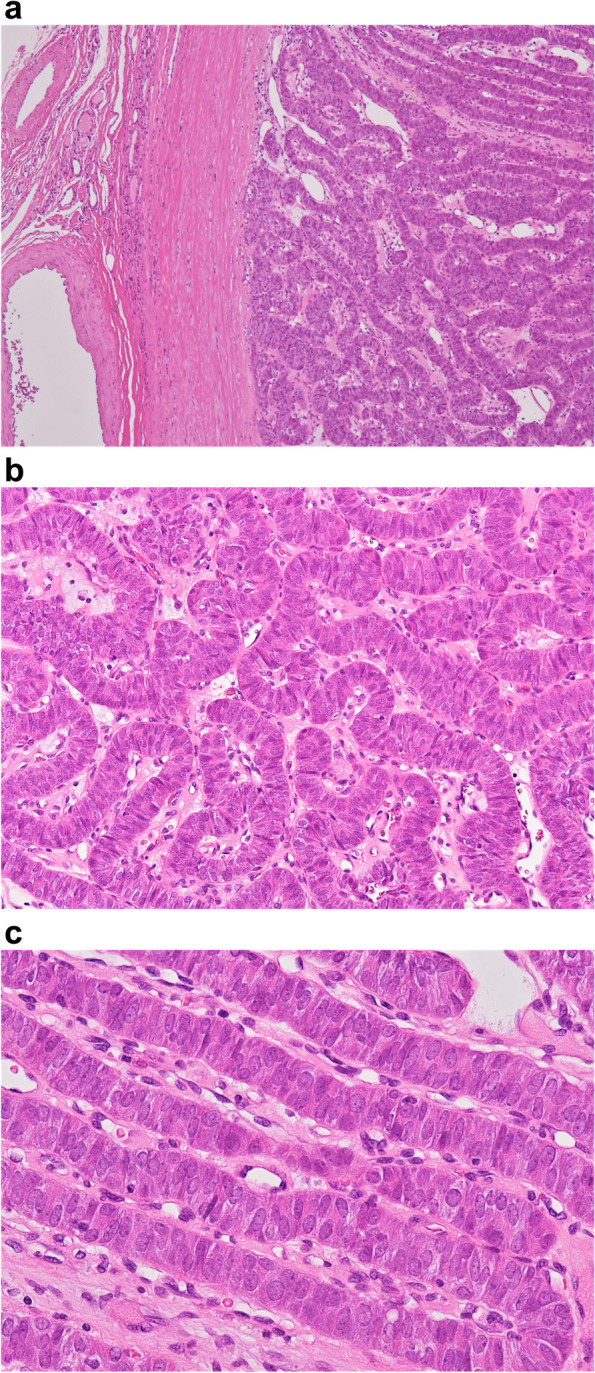
The tumor is entirely encapsulated by thick connective tissue and composed of thin and long trabeculae (**a**). The trabeculae are wiggly (**b**). Tumor cells are elongated or fusiform and arranged perpendicularly to the axis of the trabeculae (**c**)

The mass was encapsulated in a thick capsule. The cut surface was solid, whitish-yellow in color, and was associated with focal fibrosis under the capsule (Fig. 3). Microscopically, the tumor was encapsulated and did not show capsular or vascular invasion (Fig. 4a). It was composed of thin and long trabeculae, and other growth patterns, including papillary, follicular, solid, and insular, were not observed. The trabeculae were straight or wigged (Fig. 4b). Lumina and colloids were not observed. The tumor cells were elongated or fusiform and arranged perpendicular to the axis of the trabeculae (Fig. 4c). The cytoplasm was amphophilic and no yellow bodies were observed. The nuclei were oval or short spindles and did not show the nuclear characteristics of PTC, such as intranuclear cytoplasmic inclusions, irregularly shaped nuclei, ground-glass chromatin, and nuclear grooves. A thin fibrovascular stroma was intercalated between the trabeculae. Focally hyalinized or edematous stroma was observed. Amyloid deposition, necrosis, and mitosis were not observed.

**Fig. 5:**
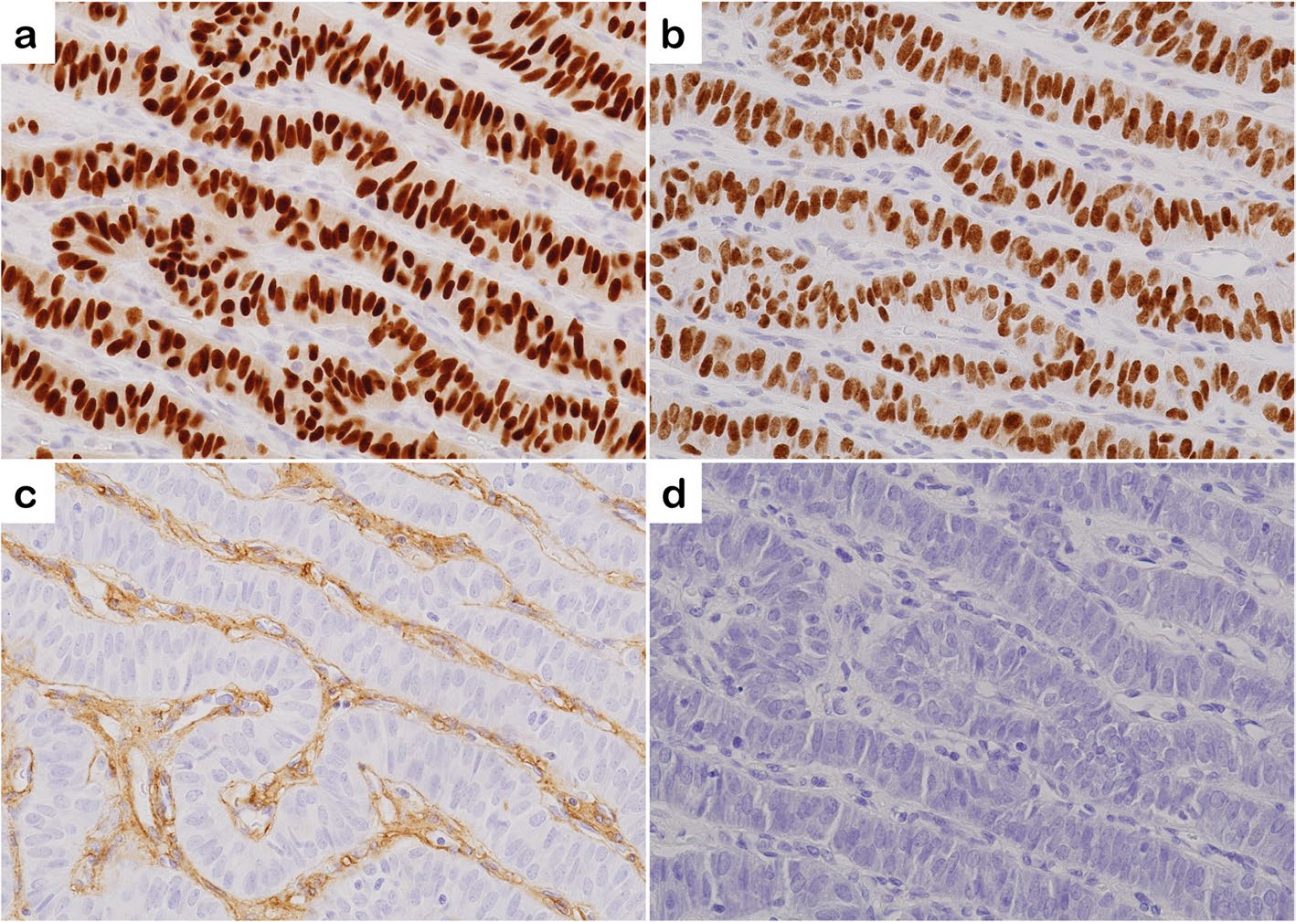
Tumor cells are positive for PAX8 (**a**) and TTF-1 (**b**) and negative for thyroglobulin (**c**). The inter- and intra-trabecular accumulation of type IV collagen-positive materials is not present (**d**)

Immunohistochemically, the tumor cells were positive for paired-box gene 8 (PAX8) (EPR13510, Abcam, Cambridge, UK) (Fig. 5a) and thyroid transcription factor-1 (TTF-1) (8G7G3/1, Dako, Carpinteria, CA, USA) (Fig. 5b). Thyroglobulin (polyclonal, Histofine, Tokyo, Japan) (Fig. 5c), calcitonin (polyclonal, Dako, Denmark, Glostrup), chromogranin A (polyclonal, Dako, Carpinteria, CA, USA), and carcinoembryonic antigen (CEA; COL-1, Histofine) were negative. Membranous reactivity of MIB-1 (MIB-1, Dako, Glostrup, Denmark) was not observed. No inter- and intra-trabecular accumulation of type IV collagen-positive materials (polyclonal; Abcam, Cambridge, UK) was observed (Fig. 5d). The Ki-67 labeling index (MIB-1, Dako, Glostrup, Denmark) was less than 1%.

In molecular testing, we analyzed common genetic alterations to FTA, FTC, PTC, HTT, PDTC, and MTC [3] using the next-generation sequencing custom targeted panel (Ion Torrent Genexus, Thermo Fisher Sci., MA, USA), including point mutations in *BRAF, N/K/H-RAS, RET, TERT* promoter*, EIF1AX, AKT1, CTNNB1, PTEN*, etc. We also analyzed the presence of *PAX8/GLIS1* and *PAX8/GLIS3* fusion genes, which are hallmarks of HTT, using RT-PCR (Nikiforova MN et al., PMID: 30,648,929). None of *PAX8/GLIS1 and PAX8/GLIS3 and mutations in BRAF, HRAS, KRAS, NRAS, TERT promoter, CTNNB1, PTEN, *and *RET* were detected.

No recurrence or metastasis was observed during the 2-year postoperative follow-up.

## Discussion and conclusions

Trabecular growth patterns have been observed in various thyroid tumors, including HTT, MTC, FTA, FTC, PTC, PDTC, intrathyroidal parathyroid adenoma/carcinoma, and paraganglioma [1]. The growth pattern is not predominant in most cases and is always associated with other growth patterns. The present case showed a trabecular growth pattern throughout the tumor and was characterized by thin and long trabeculae composed of tumor cells arranged perpendicular to their axes. This arrangement can be observed in MTC and HTT [1, 4–6].

MTC is a C cell-derived malignant tumor that produces calcitonin. Several histological variants and growth patterns of MTC have been described, with trabecular growth patterns being common [6]. Most MTCs are nonencapsulated but can be completely encapsulated as FTA [6]. In the present case, cytological findings were suggestive of MTC due to discohesiveness, naked cells, short-spindle nuclei, and granular chromatin pattern. However, the diagnosis of MTC was rejected because calcitonin production was not detected in biochemical or immunohistochemical studies. On such occasions, calcitonin-negative neuroendocrine tumors, which are extremely rare, should be considered [7], but this possibility was also denied due to the negativity of chromogranin A and synaptophysin. Furthermore, positivity for both TTF-1 and PAX8 in the present case confirmed that it was not a C-cell-derived tumor but a follicular cell origin [8, 9].

HTT is a non-invasive follicular cell-derived neoplasm characterized by a trabecular growth pattern composed of cells arranged perpendicular to the axis of the trabeculae and inter- and intra-trabecular hyalinization [4]. The growth pattern in the present case was similar to that in HTT. However, another diagnostic criterion for HTT, inter- and intra-hyalinization, was not demonstrated by immunohistochemistry using antibodies against type IV collagen [10]. In addition, other diagnostic features of HTT, such as intranuclear cytoplasmic inclusions [4], yellow bodies [11], cell membranous positivity for MIB-1 [5, 12], and tumor inferno on ultrasound examination [13], were not observed. The presence of a thick capsule is different from that of HTT, which is usually nonencapsulated [3]. Recently, Marchiò et al. described that the presence of the *PAX8–GLIS3* fusion in thyroid neoplasms may be used as an ancillary marker for the diagnosis of HTT [14]. This fusion gene was not detected in this case. Based on the above findings, it is clear that the present case did not involve HTT.

In 2015, Ohtsuki et al. reported a case of purely trabecular follicular adenoma with extraordinarily long trabeculae [2]. According to the description, the patient was a 40-year-old Japanese woman with a thyroid tumor measuring 40 × 25 × 20 mm in size. The tumor was encapsulated, had edematous portions, and was composed of long curved or folded trabeculae and did not reveal follicular structures or colloids. Tumor cells were arranged perpendicular to the axis of the trabeculae. No surrounding invasion, mitotic figure, vascular invasion, or lymph node metastasis were observed. Immunohistochemically, the tumor cells were positive for cytokeratin CAM5.2 and thyroglobulin and negative for calcitonin, CEA, and amyloid A. A partial linear pattern was observed for type IV collagen expression. MIB-1 reactivity was observed in < 1% of the tumor nuclei, with no staining of the tumor cell membranes. The histological and immunohistochemical findings were identical to those of our case except for thyroglobulin immunoreactivity. In the present case, tumor cells were negative for thyroglobulin. Regardless of the reactivity to thyroglobulin, we would consider their case and ours to be the same type of tumor derived from thyroid follicular cells. However, molecular testing was not performed by Ohtsuki et al. We attempted to identify genetic mutations characteristic of the present tumor and confirmed that none of the genetic mutations reported in FTA, FTC, PTC, HTT, PDTC, and MTC, were present. The results may suggest the possibility of tumors other than the aforementioned ones.

According to the 2022 World Health Organization Classification of thyroid neoplasm [15], encapsulated follicular cell-derived tumors without malignant features, such as nuclear findings of PTC, capsular invasion, vascular invasion, necrosis, and mitosis, were diagnosed as FTA and categorized as benign lesions. However, the histological findings were unique and differed considerably from those of FTA. No genetic mutations seen in FTA were present. We believe it is difficult to diagnose the present case without follicular growth pattern, colloid, reactivity to thyroglobulin, or *RAS* mutation as FTA. We would like to report our case as a novel disease entity called non-hyalinizing trabecular thyroid adenoma, which has the diagnostic pitfalls of HTT and MTC.

## Data Availability

Data supporting the findings of this study are available from the corresponding author on request.

## Abbreviations

FTA: Follicular thyroid adenoma
FTC: Follicular thyroid carcinoma
HTT: Hyalinizing trabecular tumor
MTC: Medullary thyroid carcinoma
PAX8: Paired-box gene 8
PDTC: Poorly differentiated thyroid carcinoma
PTC: Papillary thyroid carcinoma
TTF-1: Thyroid transcription factor-1
